# Paecilomyces variotti in deep dental caries

**DOI:** 10.4317/jced.60031

**Published:** 2022-12-01

**Authors:** Frederico-Kleinsorge Daibert, Manoel-Marques-Evangelista Oliveira, Josué-da Costa Lima-Junior, Gisela-Lara da Costa, Flávio-Rodrigues-Ferreira Alves, Lúcio-Souza Gonçalves, Fábio-Ramôa Pires

**Affiliations:** 1 Post-Graduation Program in Dentistry, Estácio de Sá University, Rio de Janeiro/RJ, Brazil; 2 Laboratory of Taxonomy, Biochemistry and Bioprospecting of Fungi, Oswaldo Cruz Institute, FIOCRUZ, Rio de Janeiro, Brazil; 3 Post-Graduation Program in Dentistry, Unigranrio, Duque de Caxias/RJ, Brazil

## Abstract

*Paecilomyces variotti* (*P. variotti*) is a fungal species found in soil, wood and some foods, and has been associated with some severe systemic infections. *P. variotti* has not been previously identified in carious tissue, and the aim of the present study is to report the presence of *P. variotti* in a deep carious lesion discussing its possible local and systemic associations. A 28 year-old male was submitted to extraction of the upper left second premolar (tooth #25) presenting a deep carious lesion. After extraction the tooth was cleaved in its long axis, and the infected dentinal tissue was curetted and submitted to microbiological analysis using CHROMagar® Candida medium and Malt Extract Agar. Macroscopic and microscopic analysis confirmed the presence of P. variotti in the carious tissue. Post-operatory period was uneventful, healing of the dental socket was complete, and the patient remained well during the follow-up period. *P. variotti*, a fungus not considered saprophyte in the oral cavity, was encountered in a deep caries lesion, and its potential association with local and systemic infections should be considered.

** Key words:**Paecilomyces variotti, dental caries.

## Introduction

Deep dental caries and endodontic infections are characterized by the participation of a vast, mixed and heterogeneous microbiota (1-3). Apart from oral microrganisms, other microbial species derived from exogenous sources, such as food, can inhabit and constitute at least part of the local microbiota in several oral microenvironments, such as dental caries (4). Fungi possess a huge survival and adaptation capacity when colonizing different habitats and, due to the modern molecular identification techniques, they have been associated with several specific clinical scenarios, including deep caries and endodontic infections (1).

*Paecilomyces variotti* is a fungal species usually found in soil, wood and some foods, and has been associated with some severe systemic infections, mostly in the upper and lower respiratory tract, and in immunocompromised individuals (5-8). The aim of the present study is to report the presence of *P. variotti* in a deep carious lesion discussing its possible local and systemic associations.

## Case Report

**Figure 1:**
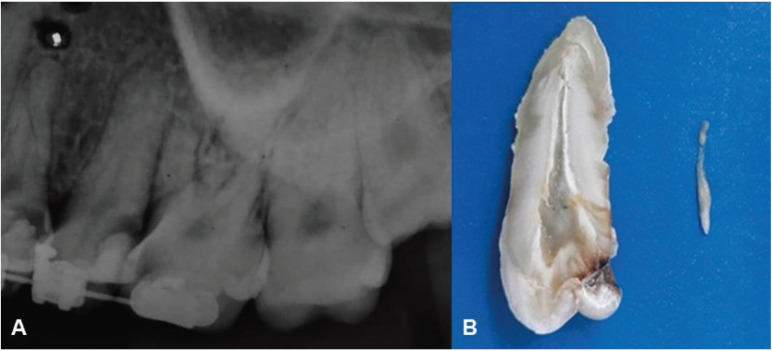
A. Periapical radiograph showing an extensive deep carious lesion on the oclusal and distal surfaces of the upper left second premolar (tooth #25). B. Upper left second premolar extracted and cleaved in the buccal-lingual orientation, showing the deep carious lesion and the pulp tissue removed.

A 28-year-old male under orthodontic treatment was referred for evaluation of the upper left second premolar (tooth #25) in January 2019. Medical history revealed sporadic mild sinusitis episodes. The patient was asymptomatic and a periapical radiograph revealed the presence of an extensive deep carious lesion on the distal surface (Fig. 1A). Pulp tests showed a positive response to thermal stimuli, with exacerbation of pain after stimuli and persistence after its removal. These findings, together with the absence of periapical bone alterations, rendered a diagnosis of irreversible pulpitis.

Extraction of the upper left second premolar was indicated and performed under local anesthesia in March 2019. After extraction, the tooth was washed in running water and cleaved in its long axis in the buccal-lingual orientation (Fig. 1B). The pulp tissue was removed and stored in 10% formalin solution and the infected dentinal tissue was curetted with an appropriate instrument and stored in Tris-EDTA for future microbiological analysis. The deepness of the carious lesion close to the pulp and the removed pulp tissue are shown in Figure 1. All procedures were performed under strict aseptic conditions inside an aseptic cabinet.

**Figure 2:**
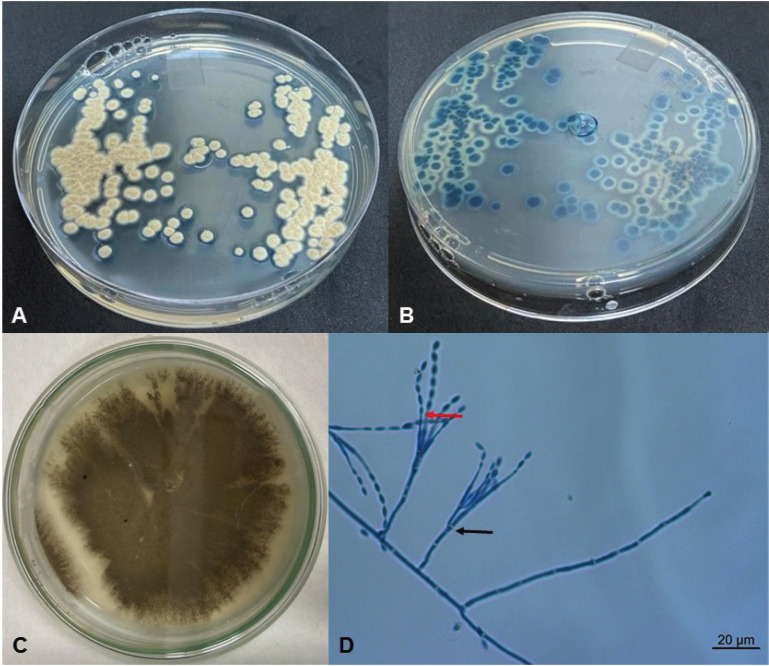
A,B) Colonies grown on CHROMagar *Candida* for 48h at 30°C (A – front, B – verse). C) *Paecilomyces variotti* in the MEA medium forming velvety olive brown colonies. D) Microscopic characteristics of *Paecilomyces variotti* with observation of irregularly branched conidiophores (black arrow), and the phialides with a broad base ending in a long and slender neck (red arrow) (magnification 400x).

The infected dentinal tissue was diluted in 2 mL of 0.9% saline solution and 2 µL of this suspension was applied to a plate containing CHROMagar® *Candida* (Difco, Becton-Dickinson and Company, USA) (Fig. 2A). The plate was incubated at 35°C for 48 hours to assess the purity and chromogenic characteristics of the sample. Macroscopic observation of the plate showed several cobalt blue colonies with cotton and rough appearance, not compatible with any *Candida* species on CHROMagar® (Fig. 2A,B). These colonies were selected for polyphasic identification species level using macro and microscopic characters in Malt Extract Agar (MEA) (Difco, Becton-Dickinson and Company, USA). Phenotypic characterization was performed by culture of the isolate in MEA for 7 days incubated in the dark at 25°C, and showed velvety olive brown colonies suggestive of *Paecilomyces variotii* (Fig. 2C). Identification was concluded by the microscopic observation of characteristic irregularly branched conidiophores and the presence of broad base ending phialides in a long and slender neck found in *P. variotii* (Fig. 2D).

Post-operatory period was uneventful and the patient did not report pain, abscess or any other discomfort in the area of the extraction. Healing was complete and, during follow-up, the patient only reported sporadic sinusitis episodes similar to those previously reported in his medical history.

## Discussion

Detection of fungal species in the oral cavity is common, but their presence in deep caries has not been regularly studied (3,9). *Paecilomyces variotti* has been encountered in several food types and its presence in a carious lesion seems not surprising. However, as deep caries are the main entrance to pulp and periapical tissues and are associated to the pathogenesis of apical periodontitis, this feature can be of local relevance. Moreover, apical periodontitis is potentially associated with regional and even systemic dissemination, as in apical abscesses and extrarradicular infection. *P. variotti* are fungi associated with emerging fungal infections and conceptually these infections develop in a dynamic interplay of altered hosts and/or permissive environmental conditions and/or selective antifungal profile of resistance.

The present case is the first demonstration of the presence of *P. variotti* in carious tissue published in literature up to know. An important result in our study was the growth of *P. variotti* for first time in CHROMagar® *Candida*, a selective medium for yeasts of *Candida* genera. Therefore, the described workflow could be useful in screening oral fungal infections by *P. variotti* in future studies. The association with molecular methods should be also encouraged for detection of additional fungus species in dental and oral and maxillofacial tissues (10). *Paecilomyces* species have been associated with several local and systemic conditions in humans, including disseminated fungal infection (11,12), urinary tract infections (13), peritonitis (5), pneumonia and other upper and lower respiratory tract diseases (6-8), sinusitis (7), and endocarditis (14). In the present case, the patient did not report any local or systemic complaints, except for mild sinusitis episodes. As the pattern of the episodes remained similar after tooth removal, it is unlikely that they would be primarily associated with *P. variotti* infection.

The fact that in the present report *P. variotti* has been found in a deep carious lesion does not mean it is involved as an etiological factor, an agent related to the evolution of the disease or a modifying agent. It is possible that *P. variotti* could be solely a colonizing agent in deep caries due to the favorable survival and growing characteristics, including temperature, humidity and nutritional factors. However, as deep carious lesions are a port of entry to the pulp tissue and to the periapical tissues, it is not possible to rule out the risks of a local and/or systemic dissemination of the fungus. As previously shown, *Paecilomyces* species have been associated with sinusitis (7), pneumonia and endocarditis (14), all of them possibly associated with oral microrganisms. In Endodontics, there is increasing awareness on the possible participation of microrganisms from the root canals from upper premolars and molars in the pathogenesis of maxillary sinusitis (15). It is also worthwhile to consider the risk of endocarditis triggered by endodontic procedures that could produce transient trans- and post-operatory bacteremia and/or fungaemia.

*Paecilomyces variotti*, a fungus that is not considered saprophyte in the oral cavity, was encountered in a deep caries lesion, and its potential association with local and systemic infections should be considered.
